# Scaling behavior of InAlN/GaN HEMTs on silicon for RF applications

**DOI:** 10.1038/s41598-022-21092-9

**Published:** 2022-10-06

**Authors:** Peng Cui, Yuping Zeng

**Affiliations:** 1 Institute of Novel Semiconductors, Shandong University, Jinan, 250100 Shandong China; 2 Department of Electrical and Computer Engineering, University of Delaware, Newark, DE 19716 USA

**Keywords:** Nanoscale devices, Other nanotechnology, Electrical and electronic engineering

## Abstract

Due to the low cost and the scaling capability of Si substrate, InAlN/GaN high-electron-mobility transistors (HEMTs) on silicon substrate have attracted more and more attentions. In this paper, a high-performance 50-nm-gate-length InAlN/GaN HEMT on Si with a high on/off current (*I*_on_/*I*_off_) ratio of 7.28 × 10^6^, an average subthreshold swing (SS) of 72 mV/dec, a low drain-induced barrier lowing (DIBL) of 88 mV, an off-state three-terminal breakdown voltage (*BV*_ds_) of 36 V, a current/power gain cutoff frequency (*f*_T_/*f*_max_) of 140/215 GHz, and a Johnson’s figure-of-merit (JFOM) of 5.04 THz V is simultaneously demonstrated. The device extrinsic and intrinsic parameters are extracted using equivalent circuit model, which is verified by the good agreement between simulated and measured *S-*parameter values. Then the scaling behavior of InAlN/GaN HEMTs on Si is predicted using the extracted extrinsic and intrinsic parameters of devices with different gate lengths (*L*_g_). It presents that a *f*_T_/*f*_max_ of 230/327 GHz can be achieved when *L*_g_ scales down to 20 nm with the technology developed in the study, and an improved *f*_T_/*f*_max_ of 320/535 GHz can be achieved on a 20-nm-gate-length InAlN/GaN HEMT with regrown ohmic contact technology and 30% decreased parasitic capacitance. This study confirms the feasibility of further improvement of InAlN/GaN HEMTs on Si for RF applications.

## Introduction

InAlN/GaN high-electron-mobility transistors (HEMTs) on silicon substrate have attracted more and more attentions due to the low cost and the scaling capability of Si substrate^1–4^. Li et al*.* demonstrated an InAlN/GaN HEMT on Si with a gate length (*L*_g_) of 55 nm and a source-drain spacing (*L*_sd_) of 175 nm^5^ using *n*^++^-GaN regrowth source/drain contacts. The device presents a maximum drain current (*I*_d, max_) of 2.8 A/mm, a peak extrinsic transconductance (*g*_m_) of 0.66 S/mm, and a current/power gain cutoff frequency (*f*_T_/*f*_max_) of 250/204 GHz. Xie et al*.* reported that a record *f*_T_ of 310 GHz was achieved on an InAlN/GaN HEMT on Si with a 40-nm gate length^6^. Cui et al*.* demonstrated an 80-nm-gate-length InAlN/GaN HEMT on Si with a record high on/off current (*I*_on_/*I*_off_) ratio of 1.58 × 10^6^, a steep subthreshold swing (SS) of 65 mV/dec, and a *f*_T_ of 200 GHz, resulting in a record high *f*_T_ × *L*_g_ = 16 GHz µm^7^. Chowdhury et al*.* demonstrated a complementary logic circuit (an inverter) on a GaN-on-Si platform with a record maximum voltage gain of 27 V/V at an input voltage of 0.59 V with *V*_DD_ = 5 V^8^. Xie et al*.* reported an InAlN/GaN HEMT on Si with a *f*_T_ of 210 GHz and a three-terminal off-state breakdown voltage (*BV*_ds_) of 46 V, leading to a record high Johnson’s figure-of-merit (JFOM = *f*_T_ × BV_ds_) of 8.8 THz V^9^. Then et al.reported the high *f*_T_/*f*_max_ of 190/300 GHz was achieved on the e-mode high-k InAlN/GaN transistor on 300 mm Si substrate^10^.

However, to the best of our knowledge, the highest *f*_T_/*f*_max_ of 454/444 GHz and 348/340 GHz were achieved on 20-nm-gate-length AlN/GaN HEMT^11^ and 27-nm-gate-length InAlN/GaN HEMTs on SiC^12^, respectively. Although excellent performances have been demonstrated, InAlN/GaN HEMTs on Si still presents much room to be improved compared with GaN HEMTs on SiC substrate. The InAlN barrier can be grown lattice-matched to GaN when the In component is 17%, which makes it easier grow than AlN on GaN^13^. The InAlN/GaN heterostructure also exhibits higher quantum well polarization-induced charge than AlGaN/GaN heterostructure, resulting in higher channel electron density and drain current^14,15^. In addition, compared with AlGaN/GaN, a thinner InAlN barrier in InAlN/GaN HEMTs not only can offer higher frequency performance with an improved device transconductance, but also can suppress the short-channel effect with the reduced gate-to-channel distance^16,17^. Hence, exploring the possible limiting factors of InAlN/GaN HEMTs on Si is significant to further improve the device performance. In this paper, high-performance InAlN/GaN HEMTs on Si are demonstrated. The extrinsic and intrinsic parameters of devices with different gate lengths are extracted and the scale behavior of InAlN/GaN HEMTs on Si is predicted. It presents that a *f*_T_/*f*_max_ of 230/327 GHz can be achieved when *L*_g_ scales down to 20 nm with the technology developed in the study, and an improved *f*_T_/*f*_max_ of 320/535 GHz can be achieved on a 20-nm-gate-length InAlN/GaN HEMTs with regrowth ohmic contact technology and 30% decreased parasitic capacitance. This confirms the feasibility of further improvement of InAlN/GaN HEMTs on Si for RF applications.

## Experiment

**Figure 1:**
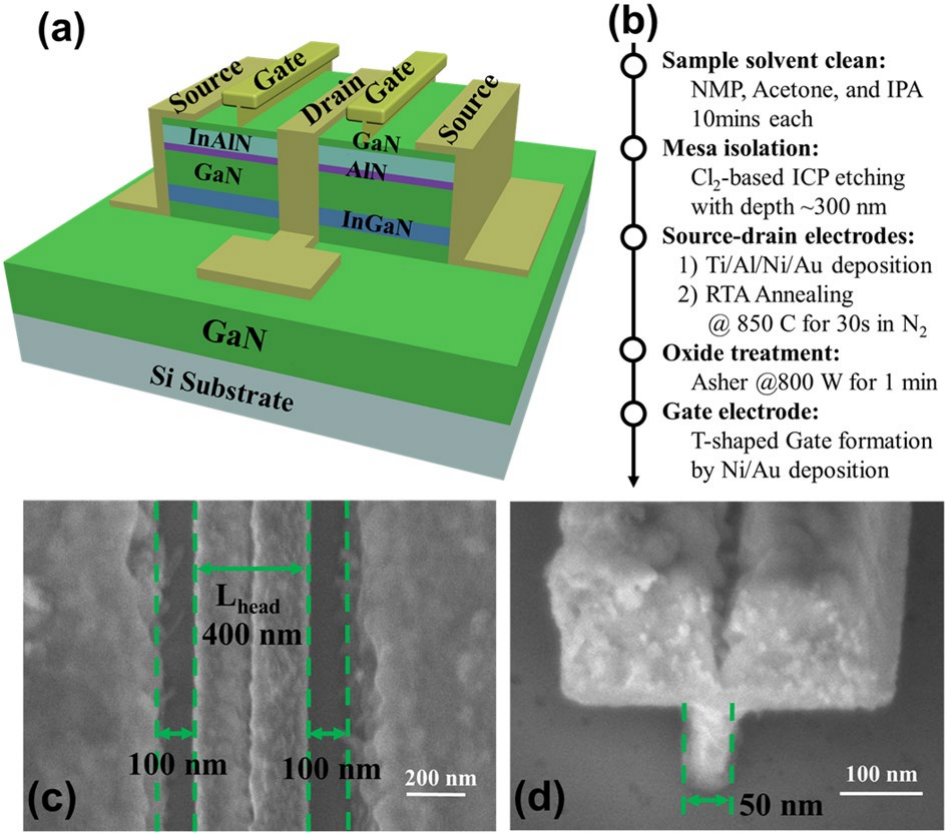
**(a)** Schematic of fabricated InAlN/GaN HEMT; **(b)** Detailed device fabrication steps; **(c)** a plan-view scanning electron microscopy (SEM) image of the InAlN/GaN HEMT with a gate head length (*L*_head_) of 400 nm and a source-drain spacing (*L*_sd_) of 600 nm; **(d)** A SEM image of T-shaped gate structure depicting a gate footprint of 50 nm.

Figure 1a shows the lattice-matched In_0.17_Al_0.83_N/GaN heterostructure, which is grown on a Si substrate by metalorganic chemical vapor deposition (MOCVD). The epitaxial layer structure consists of a 2-nm GaN cap layer, an 8-nm In_0.17_Al_0.83_N barrier layer, a 1-nm AlN interlayer, a 15-nm GaN channel layer, a 4-nm In_0.12_Ga_0.88_N back-barrier layer, and a 2-μm undoped GaN buffer layer^18^. The electron sheet concentration and electron mobility measured by Hall measurements were 2.28 × 10^13^ cm^−2^ and 1205 cm^2^/V s, respectively.

Figure 1b shows the detailed device fabrication steps. The device fabrication started with mesa isolation using Cl_2_/CH_4_/He/Ar inductively coupled plasma etching. Then Ti/Al/Ni/Au stack was deposited and annealed at 850 °C for 40 s in N_2_ to form the alloyed ohmic contacts. The ohmic contact resistance is 0.3 Ω mm. An oxygen plasma treatment was then applied to form the oxide layer on top of the InAlN layer, which can effectively reduce the gate leakage current and improve RF erformance^19–22^. Finally, a Ni/Au T-shaped gate with a gate width (*W*_g_) of 2 × 20 µm was fabricated by electron beam lithography. Figure 1c shows a plan-view scanning electron microscopy (SEM) image of the InAlN/GaN HEMT with a gate head length (*L*_head_) of 400 nm and a source-drain spacing (*L*_sd_) of 600 nm. Figure 1d shows a SEM image of T-shaped gate structure depicting a gate footprint of 50 nm.

## Results and discussion

### DC performance

**Figure 2:**
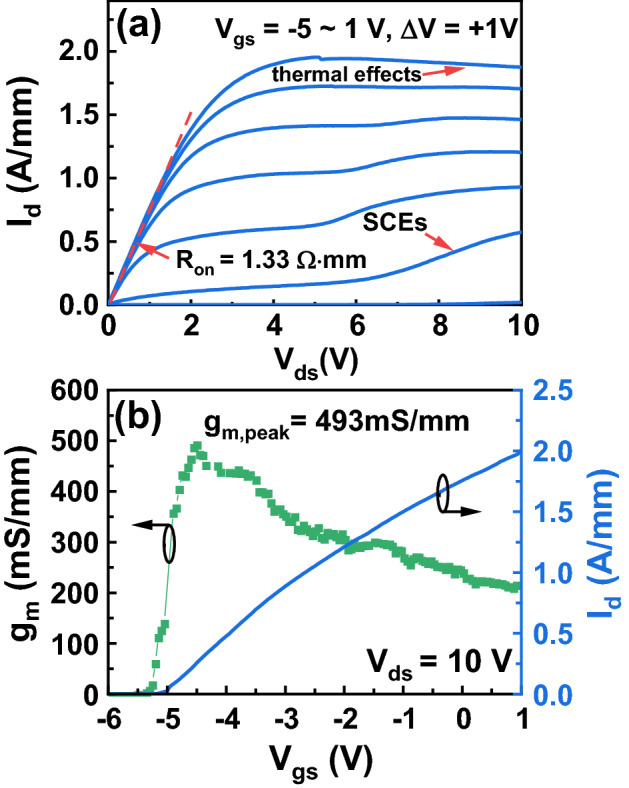
**(a)** Output characteristic, **(b)** the extrinsic transconductance *g*_m_, and the transfer characteristic at *V*_ds_ = 10 V of the InAlN/GaN HEMT with a 50-nm gate length.

The DC current–voltage (*I*–*V*) measurements are carried out by using an Agilent B1500A semiconductor parameter analyzer. Figure 2a shows the output characteristic of the InAlN/GaN HEMT with a 50-nm gate length. The device on-resistance (*R*_on_) extracted at gate-source (*V*_gs_) of 0 V and drain-source voltage (*V*_ds_) between 0 and 0.5 V is 1.33 Ω·mm. The gate-to-channel distance *t*_bar_ (including a 2-nm GaN, an 8-nm InAlN, and a 1-nm AlN) is 11 nm. Since *L*_g_ is 50 nm, the device presents an aspect ratio (*L*_g_/*t*_bar_) of 4.5. Due to the low *L*_g_/*t*_bar_, the short-channel effects (SCEs) start to appear when *V*_ds_ is larger than 5 V and *V*_gs_ is between − 4 to − 1 V. At *V*_gs_ = 1 V, drain current (*I*_d_) in saturation region presents a decrease with increased *V*_ds_, an indication of the thermal effect.

Figure 2b shows the transfer characteristic with the extracted extrinsic transconductance (*g*_m_) of the InAlN/GaN HEMT with a 50-nm gate length at *V*_ds_ = 10 V. The maximum saturation drain current (*I*_d, max_) is 2.01 A/mm at *V*_gs_ = 1 V and *V*_ds_ = 10 V. The *g*_m_ perk (*g*_m, peak_) is 493 mS/mm. To the best of our knowledge, the record high *I*_d, max_ of 2.8 A/mm and *g*_m,peak_ of 660 mS/mm were achieved on a 55-nm-gate-length InAlN/GaN HEMT on Si with regrowth technology and *L*_sd_ of 175 nm^5^. The lower *I*_d_ and *g*_m,peak_ in this study result from the regrowth-free technology and the larger source-drain spacing (*L*_sd_ = 600 nm).

**Figure 3:**
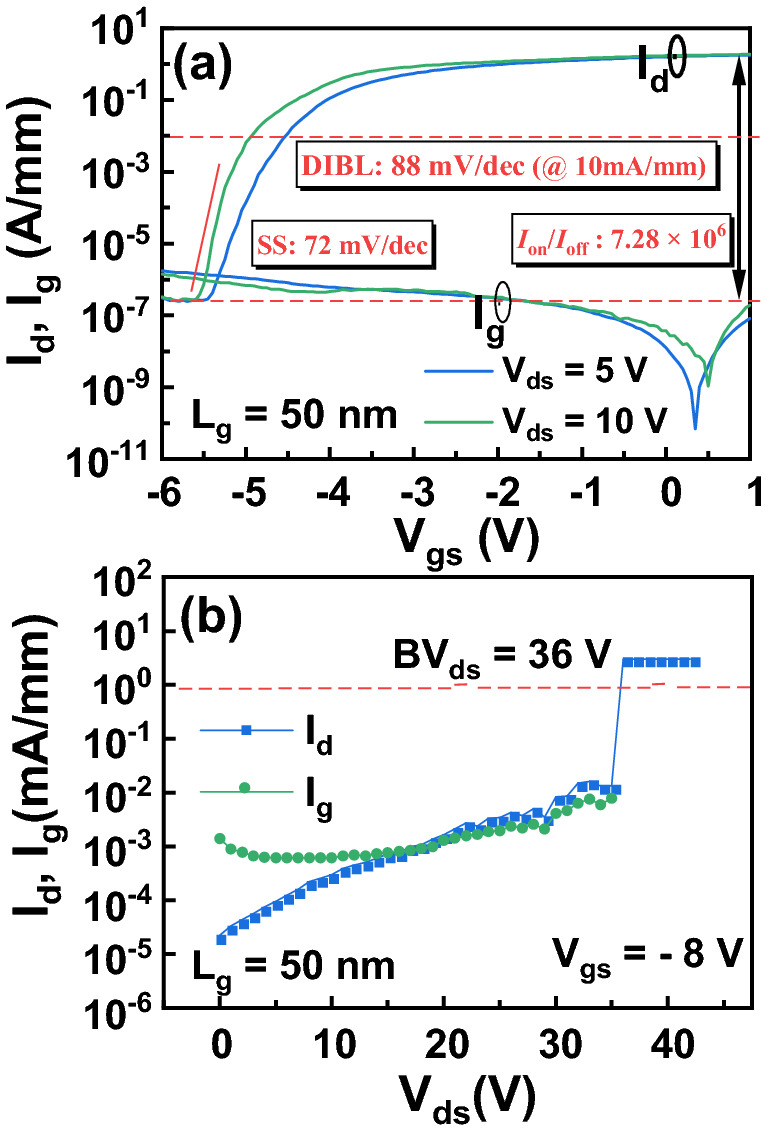
**(a)** The transfer and gate current characteristics in semi-log scale at *V*_ds_ = 10 V and 5 V, **(b)** the *I*_d_ and *I*_g_ as a function of *V*_ds_ at *V*_gs_ = − 8 V of the InAlN/GaN HEMT with a 50-nm gate length. A *BV*_ds_ of 36 V was determined.

Figure 3a shows the transfer and gate current (*I*_g_) characteristics in semi-log scale of the InAlN/GaN HEMT with a 50-nm gate length at *V*_ds_ = 5 V and 10 V, respectively. At *V*_ds_ = 10 V, the device off-current (*I*_off_) is 2.76 × 10^–7^ A/mm and the *I*_on_/*I*_off_ ratio is 7.28 × 10^6^, which are higher than the record reported values (*I*_off_ of 7.12 × 10^–7^ A/mm and *I*_on_/*I*_off_ ratio of 1.58 × 10^6^) achieved from the InAlN/GaN HEMT on Si^7^. An average subthreshold swing (SS) of 72 mV/dec over more than two orders of *I*_d_ is extracted from the transfer curve. The drain-induced barrier lowering (DIBL) of 88 mV/V is extracted at *I*_d_ = 10 mA/mm between *V*_ds_ = 10 V and *V*_ds_ = 5 V, which is the lowest value among the reported GaN HEMTs on Si. The lowest DIBL value suggests a suppressed SCEs for the sub-100 nm gate-length device. Figure 3b shows the off-state three-terminal breakdown characteristic of the 50-nm InAlN/GaN HEMT measured at *V*_gs_ = − 8 V. The device features a *BV*_ds_ of 36 V at a drain leakage current of 1 mA/mm.

### RF performance

**Figure 4:**
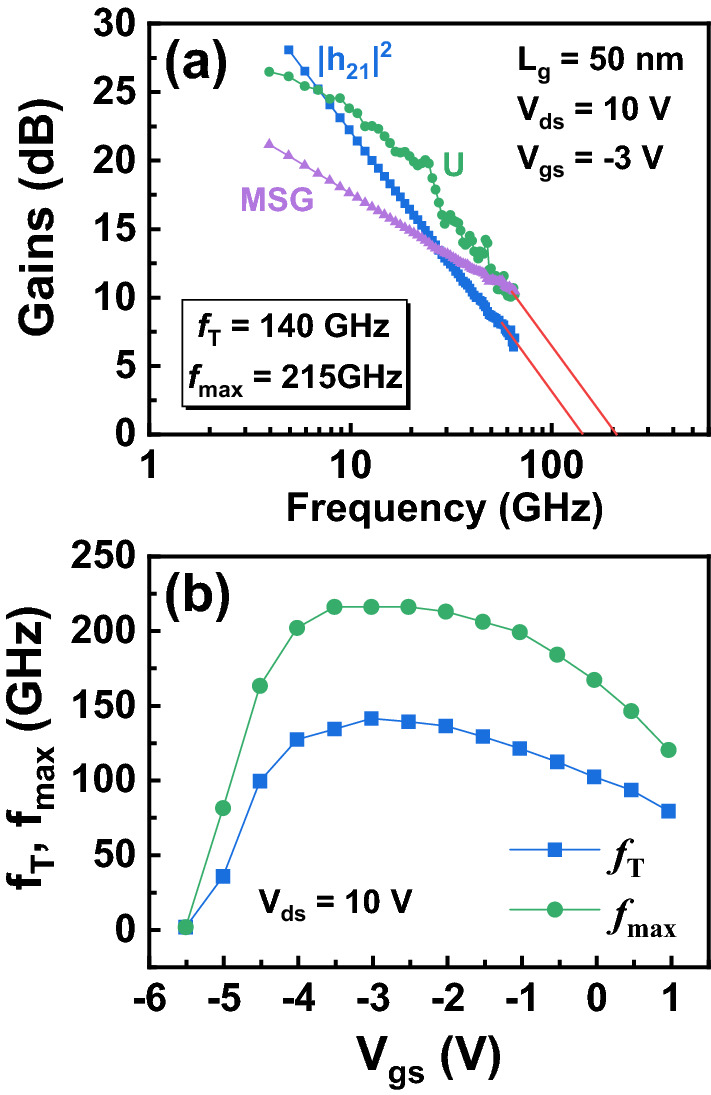
**(a)** RF performance of the InAlN/GaN HEMT with a 50-nm gate length at *V*_gs_ = − 3 V and *V*_ds_ = 10 V with *f*_T_/*f*_max_ = 140/215 GHz. **(b)** The *f*_T_ and *f*_max_ as a function of *V*_gs_.

The device RF performance is measured with a frequency range from 1 to 65 GHz. The network analyzer is calibrated using a two-port short/open/load/through method. On-wafer open and short structures is used to eliminate the effects of parasitic elements. Figure 4a shows the current gain (|h_21_|^2^), unilateral gain (U), and the maximum stable gain (MSG) as a function of frequency at *V*_ds_ = 10 V, *V*_gs_ = − 3 V after de-embedding. *f*_T_/*f*_max_ of 140/215 GHz for the InAlN/GaN HEMT with a 50-nm gate length is obtained by extrapolation of |h_21_|^2^ with a − 20 dB/dec slope. An (*f*_T_ × *f*_max_)^1/2^ of 173 GHz is obtained, which is the highest record value among the reported InAlN/GaN HEMTs on Si with regrowth-free ohmic contact technology. To the best of our knowledge, a high (*f*_T_ × *f*_max_)^1/2^ of 226 GHz (*f*_T_/*f*_max_ = 250/204 GHz) was achieved on a 55-nm InAlN/GaN HEMT on Si^5^, and a high (*f*_T_ × *f*_max_)^1/2^ of 239 GHz (*f*_T_/*f*_max_ = 190/300 GHz) was demonstrated on the e-mode high-k InAlN/GaN MISHEMTs with *L*_g_ of 50 nm^10^. The ohmic contact regrowth technology was used in both reported devices. Here for our device, the alloyed ohmic resistance (*R*_C_: 0.3 Ω mm) is higher than the reported regrowth ohmic contact resistance (*R*_C_: 0.05 Ω mm)^5^. This presents a high potential for the RF performance improvement by further decreasing the ohmic contact resistance. Due to *f*_T_/*f*_max_ of 140/215 GHz, products of *f*_T_ × *L*_g_ and *f*_max_ × *L*_g_ of 7.0 and 10.75 GHz·µm are achieved, respectively. Although neither passivation nor field plate technology is used, the 140-GHz InAlN/GaN HEMT with an *BV*_ds_ of 36 V presents a Johnson’s figure-of-merit (JFOM = *f*_T_ × BV_ds_) of 5.04 THz·V. Figure 4b shows the measured *f*_T_ and *f*_max_ of the 50-nm InAlN/GaN HEMT as a function of *V*_gs_. Both *f*_T_ and *f*_max_ show a gradual decrease compared with their peak values, presenting a good device linearity.

### Equivalent circuit model

**Figure 5:**
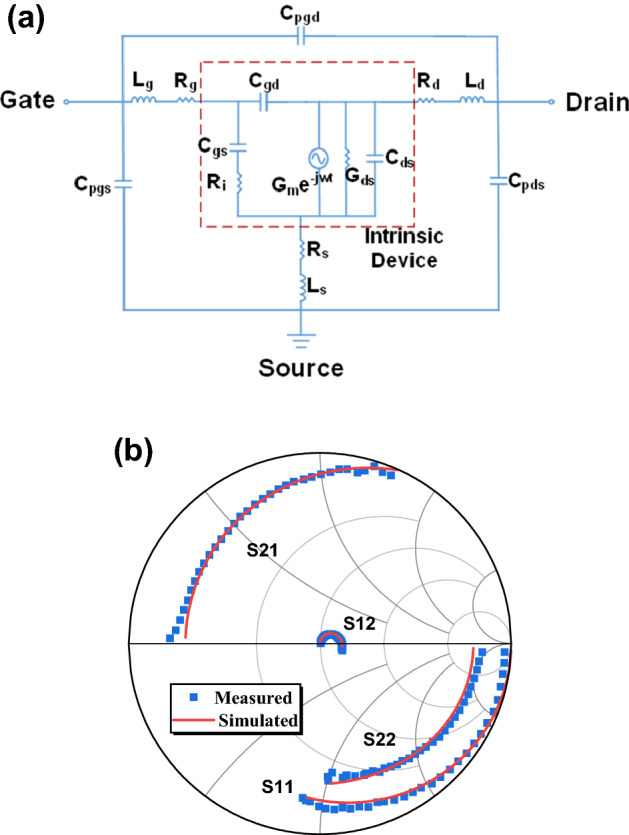
**(a)** Equivalent-circuit model for InAlN/GaN HEMT. The intrinsic elements are shown in the red dashed box. **(b)** Comparison of the simulated and measured S-parameters for the InAlN/GaN HEMT with a 50-nm gate length at *V*_ds_ = 10 V and *V*_gs_ = − 3 V.

The classical 16-element equivalent-circuit model is used for the InAlN/GaN HEMT, as shown in Fig. 5a^23,24^. Based on this model, the device extrinsic and intrinsic parameters are extracted in Table 1^23–25^. The slight discrepancy between the simulated and measured *S-*parameter values is observed in Fig. 5b, verifying the accuracy of the extracted extrinsic and intrinsic parameters. The *f*_T_ and *f*_max_ can be calculated using^23,26^

1 $$\begin{aligned} f_{T} & = \frac{{G_{m} /G_{0} }}{{2\pi ((C_{gs} + C_{gd} )(1/G_{0} + (R_{s} + R_{d} )) + (C_{gd} \cdot G_{m} /g_{0} )(R_{s} + R_{d} ))}}, \\ f_{\max } & = \frac{{f_{T} }}{{2\sqrt {(R_{s} + R_{g} + R_{i} ) \cdot G_{0} + 2\pi f_{T} R_{g} C_{gd} } }}. \\ \end{aligned}$$

where G_m_ and G_0_ are the intrinsic transconductance and drain-source conductance, respectively; *C*_gs_ and *C*_gd_ are the gate-source and gate-drain parasitic capacitance, respectively; *R*_s_, *R*_d_, *R*_g_, and *R*_i_ are the parasitic source access resistance, drain access resistance, gate electrode resistance, and input resistance, respectively.

**Table 1:** The extracted extrinsic and intrinsic parameters for the 50-nm InAlN/GaN HEMTs.

Extrinsic parameters	Intrinsic parameters
*C*_pgd_ = 1.16 fF	*C*_gs_ = 444 fF/mm
*C*_pgs_ = 26.35 fF	*C*_gd_ = 104 fF/mm
*C*_pds_ = 26.21 fF	*C*_ds_ = 318 fF/mm
*L*_s_ = 3.17 pH	*R*_i_ = 0.90 Ω mm
*L*_g_ = 44.03 pH	*G*_m_ = 573 mS/mm
*L*_d_ = 41.30 pH	*G*_0_ = 54 mS/mm
*R*_s_ = 0.43 Ω mm	*G*_m_/*G*_0_ = 10.6
*R*_g_ = 0.26 Ω mm	*τ* = 1.09 ps
*R*_d_ = 0.45 Ω mm	*f*_T, model_ = 145 GHz
	*f*_max, model_ = 218 GHz

The calculated *f*_T_/*f*_max_ = 145/218 GHz is very close to the value (*f*_T_/*f*_max_ = 140/215 GHz) extracted by the extrapolation of |h_21_|^2^ with a − 20 dB/dec slope, which confirms the excellent RF performance. The high intrinsic transconductance/drain-source conductance (*G*_m_/*G*_0_) ratio of 10.6 contributes to the high *f*_max_.

### Scaling behavior

**Figure 6:**
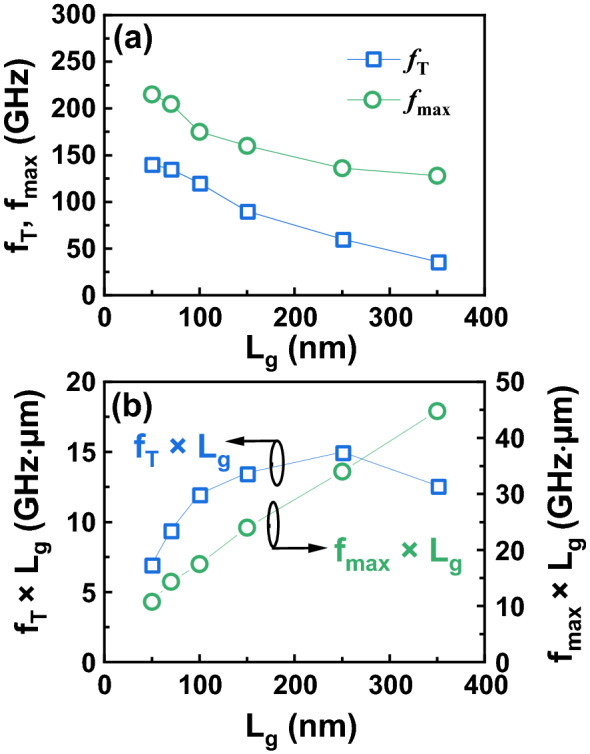
**(a)** Measured *f*_T_ and *f*_max_ as a function of *L*_g_ at *V*_gs_ = − 3 V and *V*_ds_ = 10 V. **(b)** *f*_T_ × *L*_g_ and *f*_max_ × *L*_g_ as a function of *L*_g_.

The InAlN/GaN HEMTs with *L*_g_ between 50 and 350 nm are fabricated. Figure 6a shows the measured *f*_T_/*f*_max_ of the InAlN/GaN HEMTs with different *L*_g_ at *V*_gs_ = − 3 V and *V*_ds_ = 10 V. The devices with *L*_g_ of 50, 70, 100, 150, 250, and 350 nm present *f*_T_/*f*_max_ of 140/215, 135/205, 120/170, 90/160, 60/136, 36/128 GHz, respectively. *f*_T_ × *L*_g_ and *f*_max_ × *L*_g_ are obtained in Fig. 6b. A *f*_T_ × *L*_g_ peak of 15 GHz µm is achieved on the 250-nm-gate-length InAlN/GaN HEMT with a *f*_T_ of 135 GHz. *f*_max_ × *L*_g_ presents a decrease from 44.8 GHz µm (*L*_g_ = 350 nm) to 10.75 GHz µm (*L*_g_ = 50 nm). The decrease of both *f*_T_ × *L*_g_ and *f*_max_ × *L*_g_ as *L*_*g*_ scales down means that the effect of parasitic parameters is more pronounced, thus hindering the improvement of *f*_T_ and *f*_max_. Due to the large head length of T-shaped gate (*L*_head_ = 400 nm), the transistors features higher *f*_max_ and *f*_max_ × *L*_g_.

**Figure 7:**
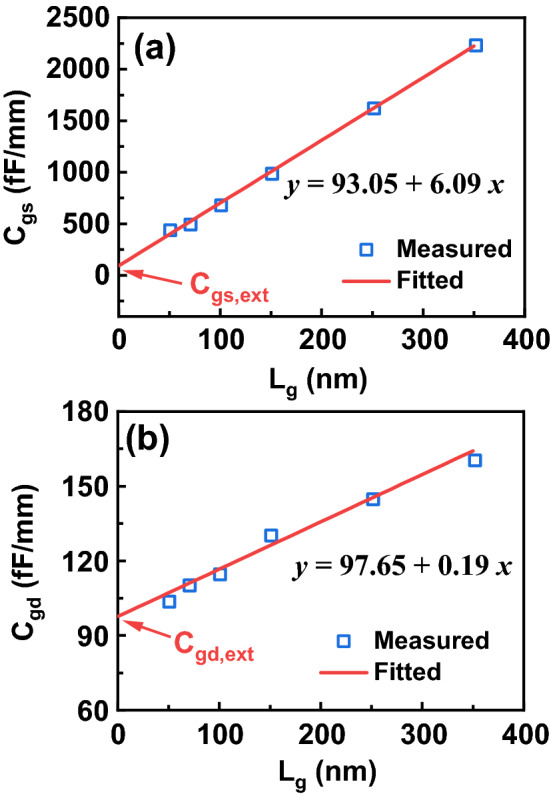
Measured and linear fitted **(a)** gate-source parasitic capacitance C_gs_ and **(b)** gate-drain parasitic capacitance C_gd_ as a function of *L*_g_ at *V*_gs_ = − 3 V and *V*_ds_ = 10 V.

To shed more light on the scaling behavior, the extrinsic and intrinsic parameters of these devices are further extracted using the equivalent circuit model discussed above. *C*_gs_ can be separated to two parts: gate-source intrinsic capacitance (*C*_gs,int_) and gate-source extrinsic capacitance (*C*_gs, ext_). It means C_gs_ = *C*_gs,int_ + C_gs,ext_^27^. *C*_gd_ can also be written as C_gd_ = *C*_gd,int_ + C_gd,ext_. Figure 7 shows the extracted *C*_gs_ and *C*_gd_ as a function of *L*_g_. Both *C*_gs_ and *C*_gd_ present a linear dependence upon *L*_g_. By linear fitting, the *C*_gs,ext_ and *C*_gd, ext_ are obtained from *C*_gs_ and *C*_gd_ at *L*_g_ = 0 nm^27^, as shown in Fig. 7. Here *C*_gs,ext_ of 93.05 fF/mm and *C*_gd,ext_ of 97.65 fF/mm are determined, respectively.

The total delay (*τ*) of transistors can be written as^27,28^

2 $$\tau = \frac{{1}}{{{2}\pi f_{T} }} = \tau_{t} + \tau_{ext} + \tau_{par}$$

Here *τ* is partitioned into three components: transit time (τ_t_), parasitic charging delay (*τ*_ext_), and parasitic resistance delay (*τ*_par_).

*τ*_t_ is the transit time under the gate region. It is related to the gate length as well as the electron velocity (*v*_e_) under the gate region, and can be calculated by^27,28^

3 $$\tau_{t} = \frac{{C_{gsi} + C_{gdi} }}{{G_{m} }} = \frac{{L_{g} }}{{v_{e} }}$$

*τ*_ext_ is parasitic charging delay through *C*_gs,ext_ as well as *C*_gd,ext_, and can be written as^27,28^

4 $$\tau_{ext} = \frac{{C_{gs,ext} + C_{gd,ext} }}{{G_{m} }}.$$

*τ*_par_ is parasitic resistance delay mainly associated with *R*_s_ as well as *R*_d_, and can be written as^27,28^

5 $$\tau_{par} = C_{gd} (R_{s} + R_{d} )\left[ {1 + \left( {1 + \frac{{C_{gs} }}{{C_{gd} }}} \right)\frac{{G_{0} }}{{G_{m} }}} \right].$$

**Figure 8:**
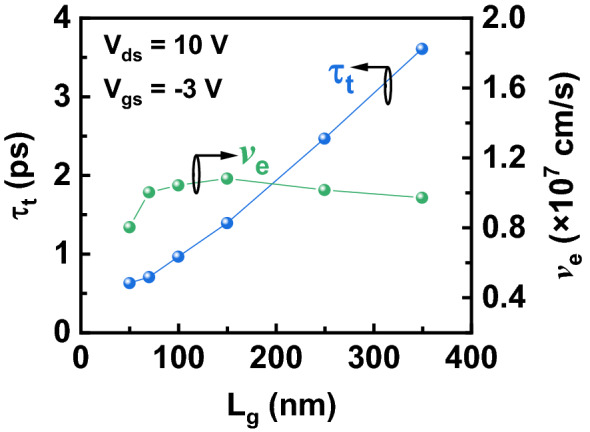
Extracted transit time (τ_t_) and electron velocity (*v*_e_) as a function of *L*_g_ at *V*_gs_ = − 3 V and *V*_ds_ = 10 V.

**Figure 9:**
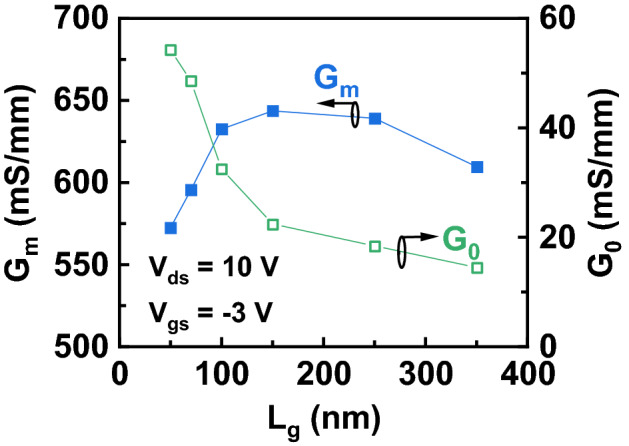
Extracted intrinsic transconductance (G_m_) and intrinsic conductance (G_0_) as a function of *L*_g_ at *V*_gs_ = − 3 V and *V*_ds_ = 10 V.

Figure 8 plots *τ*_t_ and *v*_e_ as a function of *L*_g_ calculated from (3). As *L*_g_ decreases, *τ* shows a monotonous drop, which corresponds to the increased *f*_T_. With decreased *L*_g_, *v*_e_ increases to a maximum value of 1.08 × 10^7^ cm/s (at *L*_g_ = 150 nm) and then drop to 0.80 × 10^7^ cm/s (at *L*_g_ = 50 nm). Figure 9 shows the extracted *G*_m_ and *G*_0_ from the equivalent-circuit model as a function of *L*_g_. *G*_*0*_ shows an increase with decreased *L*_g_. The dependence of *G*_m_ and *v*_e_ on *L*_*g*_ present the same trend. Based on (3), because *C*_gsi_ and *C*_gdi_ linearly depends on *L*_g_, we conclude that the change of *G*_m_ is attributed to *v*_e_ difference. The same trend of *G*_m_ and *v*_e_ on *L*_*g*_ is also observed in InAs HEMTs and result from the short channel effect^29–31^.

**Figure 10:**
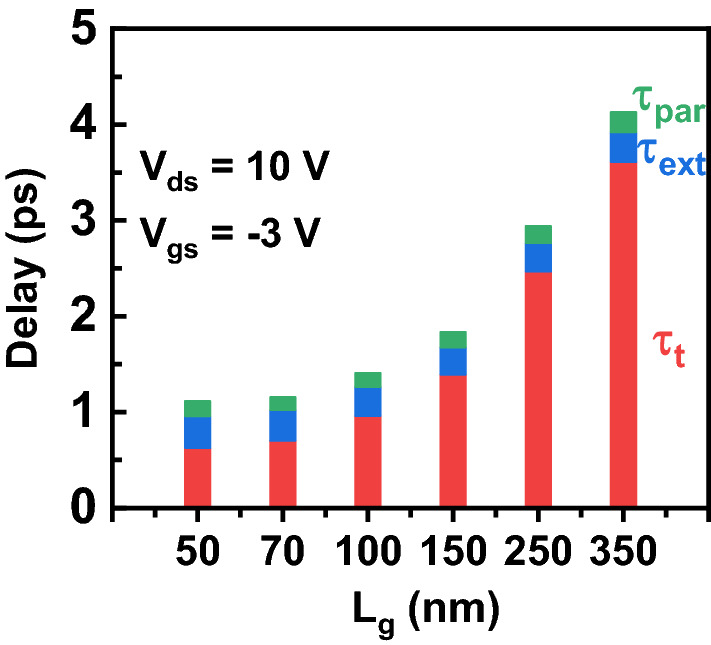
Extracted delay components as a function of *L*_g_. The delay (τ) is partitioned into three components: transit time (τ_t_), parasitic charging delay (τ_ext_), and parasitic resistance delay (τ_par_).

Figure 10 exhibits the calculated *τ*_t_, *τ*_ext_, and *τ*_par_ using (3)–(5). *τ*_ext_ and *τ*_par_ is almost unchanged. Conversely, *τ*_t_ decreases with decreased *L*_g_ and dominates the total delay in all devices. This makes it possible to decrease delay and improve *f*_T_ through downscaling of device gate length. However, for the device with *L*_g_ below 100 nm, the effect of *τ*_ext_ and *τ*_par_ become non-negligible. The ratios of (*τ*_ext_ + *τ*_par_)/*τ*_t_ are 39% and 40% for the InAlN/GaN HEMTs with *L*_g_ of 70 and 50 nm, respectively. This means the parasitic capacitance and resistance significantly hampers further *L*_g_ scaling benefits in RF performance of sub-100 nm InAlN/GaN HEMTs.

**Figure 11:**
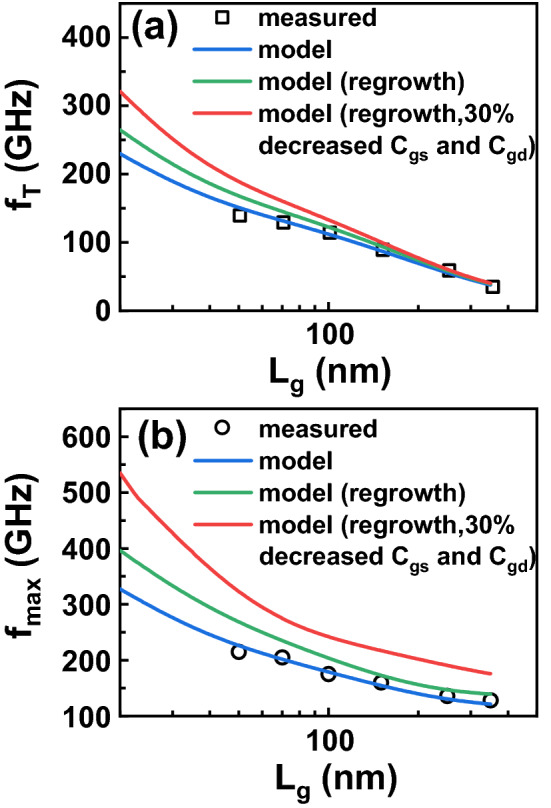
*f*_T_ and *f*_max_ under measured results (Scatters), obtained from model with extracted parameters (Blue-line), model with regrowth ohmic contact(Green-line), and model with regrowth and 30% decreased *C*_gs_ and *C*_gd_ (Red-line).

Therefore, downscaling and decreasing parasitic resistances as well as capacitances are very important for further improving device performance of InAlN/GaN HEMTs on Si. Figure 11 plots the calculated *f*_T_ and *f*_max_ based on the model and the extracted parameters (Blue-line in Fig. 11), which shows a good agreement with the measured results. In terms of the electron velocity saturation, the electron velocity of the InAlN/GaN HEMTs with *L*_g_ < 50 nm is assumed to be the same as that with *L*_g_ = 50 nm. With the obtained *v*_e_, *τ*_t_ can be obtained using (3), *τ*_ext_ is parasitic charging delay through *C*_gs,ext_ and *C*_gd,ext_, and both are the constant as shown in Fig. 7. *τ*_par_ is mainly associated with *R*_s_ and *R*_d_, which are independent on *L*_g_. As shown in Fig. 10, *τ*_ext_ and *τ*_par_ present slight change with *L*_g_. So here *τ*_par_ of the device with *L*_g_ = 50 nm is used during the model calculation. Then *f*_T_ can be calculated with the obtained *τ*_t_, *τ*_ext_ and *τ*_par_ by using (2). When *L*_g_ decrases from the 50–20 nm, the T-shaped gate head length of 400 nm is unchanged, so the effect of the small gate length variation on *R*_g_ and *R*_i_ is miminal. Hence *R*_g_ and *R*_i_ of device with *L*_g_ of 50 nm are used. *C*_gd_ is extracted from the linear fitting in Fig. 7b and then *f*_max_ is obtained using (1). The model results present that *f*_T_/*f*_max_ of 230/327 GHz can be achieved when *L*_g_ scales down to 20 nm with the technology developed in the study. To decrease the parasitic resistance, the regrowth ohmic contact can be used. Here *R*_s_ (0.30 Ω mm), *R*_d_ (0.32 Ω mm), and *G*_m_ (573 mS/mm) are changed to 0.10 Ω mm, 0.08 Ω mm, and 620 mS/mm^5^. Then new model results with regrowth technology are plotted (Green-line in Fig. 11) and a *f*_T_/*f*_max_ of 265/397 GHz is achieved on the device with a 20-nm gate length. Optimizing the detailed structure of T-shaped gate can decrease *C*_gs_ and *C*_gd_. Hence when 30% decreasing of *C*_gs_ and *C*_gd_ is added into the model, new results (Red-line in Fig. 11) are plotted and an improved *f*_T_/*f*_max_ of 320/535 GHz on 20-nm-gate-length InAlN/GaN HEMT is demonstrated. These values are comparable to the 27-nm InAlN/GaN HEMTs on SiC with *f*_T_/*f*_max_ of 348/340 GHz, suggesting the possibility of further improvement of InAlN/GaN HEMTs on Si.

## Conclusions

In summary, high-performance 50-nm InAlN/GaN HEMT on Si with an *I*_on_/*I*_off_ ratio of 7.28 × 10^6^, a SS of 72 mV/dec, a DIBL of 88 mV/V, a *BV*_ds_ of 36, a *f*_T_/*f*_max_ of 140/215 GHz, and a JFOM of 5.04 THz V are demonstrated. The extrinsic and intrinsic parameters of transistors with different *L*_g_ are extracted and the scaling behavior of InAlN/GaN HEMTs on Si is demonstrated. Based on extracted model, a *f*_T_/*f*_max_ of 320/535 GHz can be achieved on a 20-nm-gate-length InAlN/GaN HEMT with regrowth ohmic contact technology and 30% decreased parasitic capacitance. This study confirmes the feasibility of further improvement of InAlN/GaN HEMTs on Si for RF applications.

## Data Availability

The datasets supporting the conclusions of this article are included in the article.

## Abbreviations

HEMT: High-electron-mobility transistor
*I*_on_/*I*_off_: On/off current ratio
SS: Subthreshold swing
DIBL: Drain-induced barrier lowing
*BV*_ds_: Off-state three-terminal breakdown voltage
*f*_T_/*f*_max_: Current/power gain cutoff frequency
JFOM: Johnson’s figure-of-merit
*g*_m_: Extrinsic transconductance
MOCVD: Metalorganic chemical vapor deposition
SEM: Scanning electron microscopy
*I*_d_: Drain current
*I*_g_: Gate current
*L*_g_: Gate length
*L*_sd_: Source-drain spacing
*V*_gs_: Gate-source voltage
*V*_ds_: Drain-source voltage
*R*_on_: On-resistance
SCEs: Short-channel effects
|h_21_|^2^: Current gain
U: Unilateral gain
MSG: Maximum stable gain
